# Extracellular vesicles in non-small cell lung cancer stemness and clinical applications

**DOI:** 10.3389/fimmu.2024.1369356

**Published:** 2024-05-03

**Authors:** Prita Pandya, Dania S. Al-Qasrawi, Skyeler Klinge, Verline Justilien

**Affiliations:** ^1^ Department of Cancer Biology, Mayo Clinic, Jacksonville, FL, United States; ^2^ Graduate School of Biomedical Sciences, Mayo Clinic, Jacksonville, FL, United States; ^3^ Department of Biology, University of North Florida, Jacksonville, FL, United States; ^4^ Comprehensive Cancer Center, Mayo Clinic, Jacksonville, FL, United States

**Keywords:** non-small cell lung cancer, cancer stem cells, extracellular vesicles, metastasis, tumor microenvironment, biomarkers, therapeutic targeting, oncogenic signaling

## Abstract

Non-small cell lung carcinoma (NSCLC) accounts for 85% of lung cancers, the leading cause of cancer associated deaths in the US and worldwide. Within NSCLC tumors, there is a subpopulation of cancer cells termed cancer stem cells (CSCs) which exhibit stem-like properties that drive NSCLC progression, metastasis, relapse, and therapeutic resistance. Extracellular vesicles (EVs) are membrane-bound nanoparticles secreted by cells that carry vital messages for short- and long-range intercellular communication. Numerous studies have implicated NSCLC CSC-derived EVs in the factors associated with NSCLC lethality. In this review, we have discussed mechanisms of EV-directed cross-talk between CSCs and cells of the tumor microenvironment that promote stemness, tumor progression and metastasis in NSCLC. The mechanistic studies discussed herein have provided insights for developing novel NSCLC diagnostic and prognostic biomarkers and strategies to therapeutically target the NSCLC CSC niche.

## 1 Introduction

Lung cancer, an aggressive and heterogeneous disease, is the second most diagnosed cancer and the leading cause of cancer associated deaths worldwide (1). Lung cancer is broadly grouped into two major histological types, non-small cell lung cancer (NSCLC) and small cell lung cancer, which account for approximately 85% and 15% of total diagnosed cases, respectively (2). NSCLC is further classified into three main subtypes, adenocarcinoma (LUAD), squamous cell carcinoma (LUSC), and large cell carcinoma which exhibit distinct histopathology, cells and site of origin, and molecular profiles (3). Despite major advances in NSCLC treatment and diagnosis including the introduction of molecular targeted therapies, immunotherapies and low-dose computed tomography screening, the 5-year survival rate for NSCLC is at a low 25% across all stages and 8.2% for patients with metastasis (1). These poor survival rates are attributed to several factors including the frequent diagnosis of NSCLC cases in advanced or metastatic disease stages without curative treatment options. In addition, over half of NSCLC patients that present with surgically resectable disease at diagnosis may experience a recurrence of their disease post-operatively (4). Furthermore, advanced stage NSCLCs may exhibit significant initial response to molecular targeted and immuno-therapies, however, subsequent resistance frequently occurs (5), and only a small proportion of patients demonstrate sustained benefits from these therapies.

It has become apparent that not all the cancer cells within an individual tumor are equally capable of maintaining and propagating the tumor and generating metastases. This observation has given rise to the cancer stem cells (CSC) hypothesis which attributes cancer relapse, therapeutic resistance, and establishment at metastatic sites to a small subpopulation of cancer cells with stem-like properties. These ‘cancer stem cells’, ‘tumor initiating cells’ or ‘tumor propagating cells’ express stem cell associated markers and exhibit the ability to self-renew; expand and differentiate into heterogeneous lineages; and serially initiate and maintain tumors. CSCs reside in niches and communicate with surrounding differentiated cancer and stromal cells of the tumor microenvironment (TME) by a variety of signaling mechanisms including through the exchange of cell-secreted, membrane-bound nanoparticles called extracellular vesicles (EVs). CSC-derived EVs carry bioactive cargo molecules such as proteins, nucleic acids, lipids, metabolites and carbohydrates that can reflect the molecular and functional characteristics of CSCs (6) and support tumor survival by promoting treatment resistance, angiogenesis, EMT, metastasis, stemness, and modulation of the immune TME. Furthermore, reciprocal exchange of EVs from the TME support the maintenance of the CSCs. This review aims to provide an overview of the role of EVs in mediating an enhanced tumorigenic phenotype in NSCLC with an emphasis on CSC- and TME-derived EVs in NSCLC stemness, progression and metastasis formation. In addition, we will discuss implications for EVs in NSCLC diagnosis and therapeutic targeting.

## 2 CSCs in NSCLC

Tumor cells are heterogeneous, with subpopulations exhibiting distinct genotypic and phenotypic characteristics that result in divergent biological behaviors. Currently, there are two proposed theories to explain tumor cell heterogeneity: clonal evolution and CSCs (7). According to the clonal evolution theory, tumor cells continually accumulate stochastic mutations, resulting in the outgrowth of heterogeneous clone populations which are subjected to expansion and contraction based on the growth survival benefits offered by the mutations they harbor. The CSC theory proposes that tumors are organized hierarchically like healthy tissues with a stem-like sub-population at the top. The CSC population is maintained through self-renewal and can also undergo asymmetric cell division to generate differentiated progenies, giving rise to the heterogeneous bulk tumor cell population (7, 8). In 1994, Dick and colleagues were the first to report the discovery of a small subpopulation of cells in human acute myeloid leukemia with characteristics of CSCs. These leukemia CSCs expressed cell surface markers associated with normal hematopoietic stem cells and were able to initiate and propagate leukemia when engrafted into immunodeficient mice (9). Since then, similar stem-like cell populations with tumor initiating potential have been identified in solid tumors including cancers of the brain (10), pancreas (11), colon (12), skin (13), ovaries (14) and lung (15).

### 2.1 NSCLC CSC populations

Although the origin of CSCs is still debated, NSCLC CSCs are speculated to arise from distinct regional adult stem cell populations of the lung based on studies performed in genetically engineered mouse models (GEMMs). Lung basal cells of the proximal airway possess the ability to self-renew and initiate a program of differentiation to give rise to the upper airway epithelium. Tumor initiation coupled with specific gene expression and lineage tracing in GEMMs has provided compelling evidence that transformed lung basal stem cells are the origin of CSCs in LUSC (16). Alveolar type II epithelial cells in the distal lung, bronchioalveolar stem cells at the bronchioalveolar duct junction and club cells that line the bronchial airways have been reported as possible cells of origin for LUAD CSCs, due to their regional distribution, stem properties and tumor-initiating potential (17). Further compelling evidence to support hierarchy in the tumorigenicity of NSCLC cells comes from a recent study that demonstrates that the heterogenous transcriptional states of LUAD tumors occurs through a common transitional, high-plasticity cell state (HPCS) (18). The identified LUAD HPCS cells emerge rapidly during tumorigenesis, persist in advanced tumors and display high capacity for differentiation and proliferation. Furthermore, the HPCS harbors high tumorigenic potential, confers drug resistance, and associates with poor patient prognosis. The concept of plasticity has also been used to support the alternative hypothesis that CSCs may originate from more differentiated progenitor cells which have acquired the capacity for self-renewal or differentiated cancer cells (non-CSCs) that become stem-like due to mutations or influences from the microenvironment (19). Indeed, cancer associated fibroblasts were shown to induce de-differentiation of non-CSCs to CSCs via IL-6 signaling (20). Given the heterogenous nature of NSCLC, it is likely that multiple CSC populations exist with the subtypes of NSCLC harboring CSCs with unique markers and molecular drivers. The use of single cell RNA-seq to analyze tumor cell populations at the single-cell level may provide further insight into the heterogeneity of NSCLC CSCs, at least, at the transcriptional level.

CSC populations have been identified in NSCLC based on their ability to exclude the dye Hoechst 33342 (21), grow in the presence of chemotherapies (22), or express specific stem-associated markers (7). There is a growing list of cell surface markers that have been identified and used to isolate NSCLC CSC populations, including CD166, EpCAM, CD90, CXCR4, CD117, CD44, CD87, CD24, ABCG2, and CD133 (15, 22–30). Studies using a mouse model of oncogenic *K-ras-*driven LUAD have also identified a CD24+ ITGB4+ and Notch^hi^ cell population that has tumor propagating capacity, which may be relevant to human LUAD disease (31, 32). NSCLC CSCs also exhibit high expression of stem cell-associated transcription factors such as Sex-determining region Y-box 2 (SOX2), Octamer binding transcription factor 4 (OCT4), NANOG, and BMI-1 (7). Aldehyde dehydrogenase (ALDH1) activity, which is well characterized for its association with stem cell properties has been used to identify NSCLC CSC populations (33). Additional markers reported and used for lung CSC isolation are summarized in **Table 1**. NSCLC cells exhibiting stem-like properties can be enriched by growing the bulk tumor cell population in defined media under low adherence conditions as spheroids (39). In the absence of a universal marker for the detection of NSCLC CSCs, the use of this functional enrichment may allow for the selection of highly tumorigenic stem-like cells. A combination of marker expression and functional characterization is needed for robust investigation of NSCLC CSCs.

**Table 1:** List of reported NSCLC cancer stem cell markers.

Classification	Markers	Function	Tumor promoting role	References
Surface markers	CD133	Transmembrane glycoprotein	Cell adhesion, proliferation, migration, drug resistance	(15, 26, 28, 34)
	CD44	Transmembrane glycoprotein	Cell differentiation, migration, and proliferation	(27, 35)
	CD90	Glycosylphosphatidylinositol (GPI)-anchored glycoprotein	Cell-Cell and Cell-matrix interactions	(25)
	CD166/ ALCAM	Transmembrane glycoprotein	Cell differentiation, angiogenesis, stem cell maintenance	(23)
	CD117	Tyrosine kinase growth factor receptor	Sphere formation, maintenance of self-renewal.	(22)
	CD24	Glycosylphosphatidylinositol-anchored receptor	Regulation of cell migration, invasion and proliferation.	(29, 31)
	EpCAM	Transmembrane protein	Cell adhesion, differentiation, migration, drug resistance	(24, 36)
	ABCG2	ATP-binding cassette (ABC) transporter protein	Promotes multi-drug resistance	(30, 37)
	CXCR4	Chemokine surface receptor	Cell proliferation, angiogenesis, metastasis, radiation resistance	(26)
Intracellular markers	ALDH1	Enzyme catalyzing aldehyde oxidation	Multi -drug resistance	(33, 38)
	SOX2	SRY-related HMG box family (SOX) transcription factor	Cell proliferation, EMT, stem cell maintenance	(7, 38)
	OCT4/ POU5F1	POU family transcription factor	Cell pluripotency, metastasis, drug resistance	(7, 38)
	NANOG	DNA binding homeobox transcription factor	Cell differentiation, proliferation	(7, 38)
	BMI-1	Ring finger protein, a component of polycomb group complex 1	Prot-oncogene that regulates tumor suppressor PTEN function	(7)

### 2.2 CSCs in NSCLC tumorigenicity

**Figure 1:**
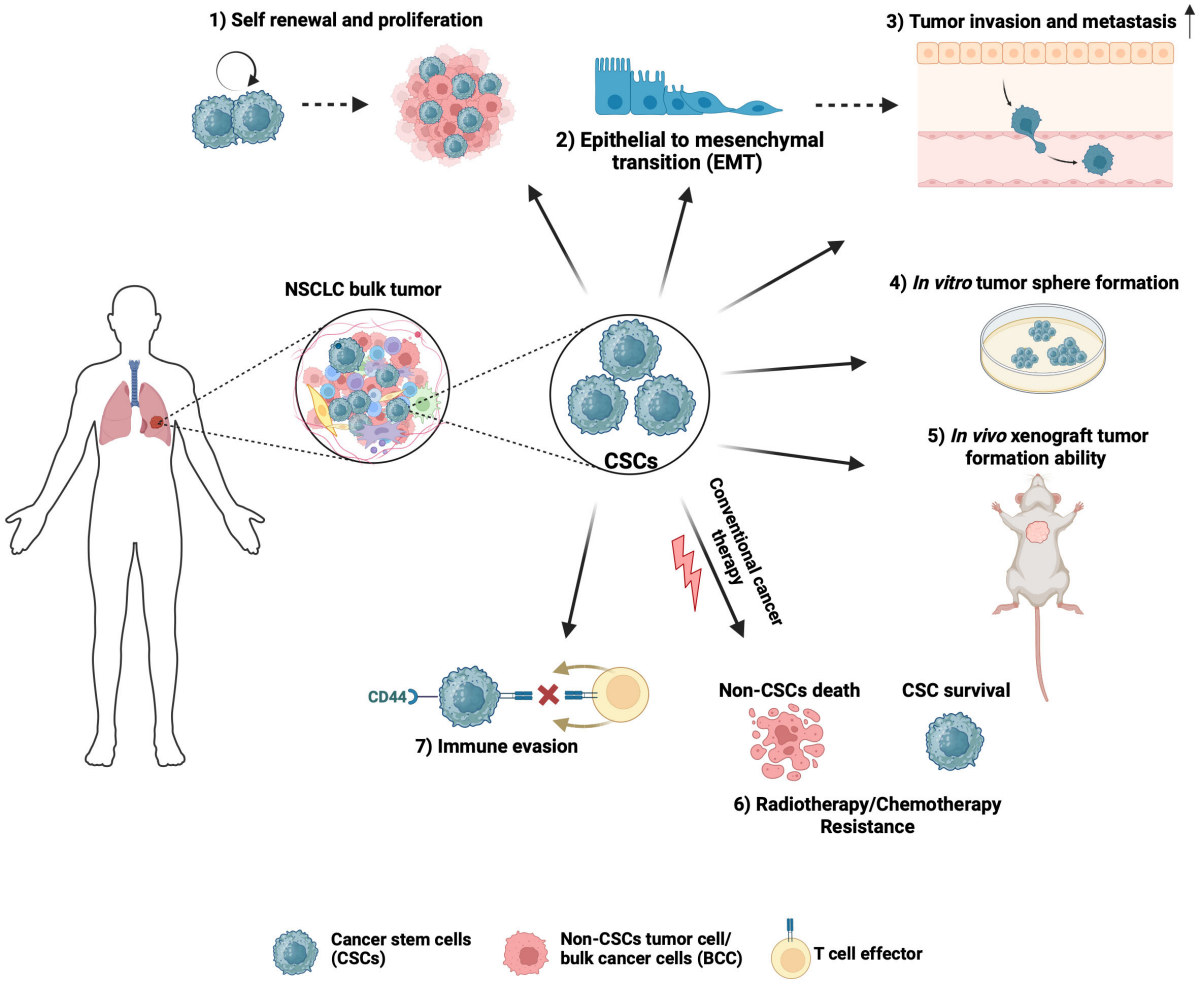
Hallmark characteristics of NSCLC CSCs that drive tumor biological processes. 1) CSCs can propagate tumors due to their self-renewal and proliferation ability. 2) The self-renewal and high plasticity of CSCs are thought to be associated with epithelial-to-mesenchymal transition. 3) CSCs have a higher propensity to invade, which is important to drive tumor progression beyond the primary site. 4) Stem-cell-associated signaling pathways allow a single CSC to proliferate and form tumor spheres *in vitro*. 5) CSCs can initiate and propagate tumors with characteristics of the original tumor. 6) Due to intrinsic ability to stay in a quiescent state, efficiently repair DNA damage, manage redox stress, and express multi-drug transporters, CSCs contribute to resistance to chemotherapy and radiation therapy, which often results in NSCLC tumor relapse. 7) CSCs inhibit immune cell activation to generate an immunosuppressive environment and contribute to immune checkpoint therapy failure. Created with BioRender.com.

CSCs exhibit characteristics that drive tumor biological processes contributing to NSCLC lethality (**Figure 1**). Regardless of the isolation or enrichment method, a major functional hallmark phenotype of CSCs is their ability to initiate and propagate tumors with characteristics of the original tumor. NSCLC CSCs isolated based on their expression of CD133 or CD44, Hoechst 33342 dye exclusion, high ALDH1 activity or spheroid growth have been found to display enhanced tumorigenicity in transplantation assays *in vivo* when compared to differentiated or bulk cancer cells (15, 27, 33, 40). The clinical relevance of NSCLC CSCs is highlighted by their intrinsic and acquired resistance to conventional cytotoxic therapeutic agents commonly used to treat NSCLC cancer, including cisplatin, etoposide, doxorubicin, and paclitaxel, as well as radiation and immunotherapies (34, 41–43). Furthermore, a possible role for CSCs in contributing to tyrosine kinase inhibitor resistance in NSCLC has been reported (44). Characteristics of CSCs that are thought to play a role in therapeutic resistance include their ability to exist in a quiescent state, efficiently repair DNA damage, manage redox stress, and express multi-drug transporters (35, 37, 45, 46). CSCs that persist after treatment have been shown to be important for NSCLC relapse (47).

CSCs have been implicated as a driver of NSCLC metastasis. During metastasis, most disseminated cancer cells do not survive, leaving a minute population of cancer cells (48). These disseminated cells with metastatic capabilities are thought to be pluripotent CSCs. Upon arrival at the distant site, disseminated CSCs may enter a period of latency, then may undergo reactivation and formation of metastatic tumors (49), resembling the parent tumor. Compelling evidence has linked CSCs to epithelial-mesenchymal transition (EMT) and cellular invasion, two essential processes in cancer metastasis. Due to their plasticity, CSCs can transition to an EMT state that promotes invasion and metastasis and a state of mesenchymal-epithelial transition during colonization at secondary sites (50). Furthermore, CSCs have a higher propensity to invade, which is important to drive tumor progression beyond the primary site (51, 52). To be able to induce tumors or to metastasize to distant organs, CSCs must evade host immune surveillance. Consistent with this notion, a higher percentage of CD133^+^/EpCAM^+^ CSCs that express PD-L1 were observed in the lymph nodes of NSCLC patients with lymph node metastasis when compared with patients without lymph node metastasis (36). Furthermore, a positive association between the stem-marker CD44 and PD-L1 was reported in NSCLC (53). CSCs may also generate an immunosuppressive, pro-tumorigenic immune environment by inhibiting the activity of various immune cells capable of killing tumor cells, such as infiltrating cytotoxic natural killer cells and CD8+ T cells (54, 55).

### 2.3 Stem cell signaling pathways in NSCLC CSCs

Stem signaling pathways, including the three main developmental pathways, Notch, Wnt, and Hedgehog (Hh), are commonly deregulated in NSCLC CSCs and play important roles in their stemness and survival. Notch signaling is critical for normal stem cell function in the lung (56), and its abnormal activation can stimulate the growth and metastasis of NSCLC cells (56). NOTCH expression and pathway activity is often used as a marker of stemness in NSCLC (57, 58). Furthermore, various NOTCH isoforms have been implicated in the stem-like properties of NSCLC CSCs. For example, NOTCH1 was reported to control the self-renewal of NSCLC CSCs and provide protection from cisplatin-induced cell death (41). Importantly, targeting Notch signaling with gamma- secretase inhibitors suppressed the growth and chemoresistance of CD133^+^, CD44^+,^ or ALDH^+^ cells (35), suggesting a functional requirement for Notch activity in NSCLC CSCs.

Hh signaling activates a transcriptional program that regulates airway epithelial progenitors during embryonic lung development and in the repair of acute airway epithelial injury (59). Aberrant Hh pathway signaling is important for the maintenance and function of NSCLC CSCs. For example, in LUSC, the two oncogenes, *PRKCI* and *SOX2* cooperate to drive a stem-like phenotype by establishing a cell-autonomous Hh signaling axis (39). CD133^+^ NSCLC CSCs secrete sonic hedgehog ligand, and Hh pathway inhibition in CD133^+^ cells decreased sphere formation, suggesting that an autocrine Hh signaling is involved in CD133^+^ CSC maintenance (60). There is also evidence to suggest that paracrine Hh signaling maybe involved in NSCLC CSC maintenance. For example, cancer-associated fibroblasts can secrete Hh ligand which mediates Hh pathway activation in NSCLC cells to promote CSC maintenance and induction of metastatic properties in NSCLC (61).

Wnt/β-catenin signaling, which regulates cell differentiation, polarity, and proliferation (62) plays an important role in regulating the activity of CSCs in a variety of cancers including NSCLC. Wnt signaling components are associated with the expression of stem marker genes in NSCLC CSCs (63). Wnt signaling may contribute to the reprogramming and maintenance of CSC states that are activated by EMT by inducing activation of a β-catenin/Twist1/TCF4 complex that binds to the promoters of CSC-related genes (64, 65). Furthermore, NSCLC CSCs have been found to be sensitive to the growth inhibitory effects of Wnt pathway inhibitors (66). The Wnt pathway ligand, Wnt5a, can increase the stem properties of ALDH^+^ CSCs in cisplatin-resistant NSCLC (66), suggesting that Wnt signaling is a mechanism that mediates the resistance of NSCLC CSCs to chemotherapies.

## 3 CSC-derived EVs in NSCLC

EV secretion was first reported in sheep reticulocytes, and initially described as a mechanism for the removal of membrane proteins during differentiation (67). Since then, EVs have become recognized as an important mechanism of intracellular communication. EVs are secreted by almost all cell types and carry membrane-enclosed molecular cargo that can mediate genetic, epigenetic and phenotypic changes in receiving cells in an autocrine or a paracrine manner (68). Secreted EVs can travel through body fluids and can therefore propagate signals at both short and long distances *in vivo* (69). EVs are involved in many essential physiologic processes in normal organism development and homeostasis and have been reported to mediate pathological conditions including cancer (70, 71).

### 3.1 EV classification, biogenesis, and cargo

Cells secrete a heterogeneous population of EVs. Current criteria to distinguish between the diverse EV populations include size, subcellular origin, function, molecular cargo and mechanism of release (72). Among the subtypes of EVs, the most studied are exosomes which have a diameter range in size of 30–150 nm (69). Exosomes originate from the late endosomal trafficking machinery, are gathered intracellularly into multivesicular bodies (MVBs) and shed upon MVB fusion with the plasma membrane (73). Ectosomes, apoptotic bodies, and large oncosomes represent additional subpopulations of EVs (~500–2000 nm) that share the feature of being secreted by budding from the cell plasma membrane and may possess different types of molecular components (71). Lastly, microvesicles (MV) are smaller particles of heterogeneous size (100–1000 nm) that do not rely on exocytosis, but rather are produced by outward germination and fission of the donor cell plasma membrane (74). The EV species rely on different mechanisms for their biogenesis. For example, exosome biogenesis is driven by the four endosomal sorting complexes (ESCRT-0–III) and other supporting factors such ALG-2-interacting protein X (ALIX) and vacuolar protein sorting-associated protein (VPS4) (70). Exosome biogenesis may also occur through an ESCRT-independent manner that involves sphingomyelinase-mediated ceramide formation and activity of ADP ribosylation factor 6 (ARF6) and phospholipase 2(PLD2) (70). The transport and fusion of MVBs to the plasma membrane is mediated by various Rab GTPases such as RAB7A, RAB11, RAB27B, and RAB35 and SNARE (soluble N-ethylmaleimide-sensitive factor attachment protein receptor) protein complexes (6). MV formation and release is mediated by an ARF6/PLD/ERK/MLCK axis and RHOA-dependent rearrangement of the actin cytoskeleton for plasma membrane germination (75, 76). Lastly, apoptotic bodies are formed in the process of apoptosis, and may contain nuclear, protein, and even organelle components from apoptotic cells (77). Since there can be an overlap in size between subtypes of EVs and there is no consensus yet on the markers assigned to specific EV subtypes based on their biogenesis, the Minimal Information for Studies of Extracellular Vesicles (MISEV) instead recommends using more general terms to describe EVs based on the size of particles such as small EVs (sEVs; <100-200nm) and medium/large EVs (m/lEVs; >200nm) (72).

EV cargo content is highly heterogeneous and reflects the molecular profile of the donor cell (70). The presence of single-strand DNA, double-strand DNA and mitochondrial DNA has been reported in EVs, although DNA is most frequently observed in larger EVs (70). RNA is by far the predominant nucleic acid in EVs and consist of mRNA and non-coding RNAs (ncRNAs). Various subtypes of ncRNAs have been detected including long non-coding RNA (lncRNA), microRNA (miRNA), ribosomal RNA (rRNA), transfer RNA (tRNA), circular RNA (circRNA), small nuclear RNA (snRNA), small nucleolar RNA (snoRNA), and piwi-interacting RNA (piRNA) (70). Large scale proteomic analyses of EVs have demonstrated that they carry a common set of protein components involved in their biogenesis, structure and putatively, their interaction with target cells (6). Unique subsets of proteins have also been identified in EVs released by different cell types (70). EVs also carry lipids such as diacylglycerol (DAG), sphingomyelin (SM), and ceramides, which are involved in the regulation of cell energy homeostasis as well as pathways that contribute to tumorigenesis (6). The loading of cargo into EVs is thought to occur through a regulated, and poorly understood, sorting mechanism that allows for discrimination between molecules that can mediate intercellular signaling. Many factors can influence the nature and abundance of EV production, cargo and release including intrinsic and extrinsic stimuli and pathological conditions such as cancer (69).

### 3.2 Regulation of CSC EV secretion

EV biogenesis and secretion is increased in cancer cells in comparison to healthy cells (78). Enhanced EV secretion may be explained by the increased activity of EV regulators. For example, components of the ESCRT complexes, SNARE proteins, syntenin, heparanase and small Rab GTPases (such as RAB27B) are overexpressed in various cancers including NSCLC (79–81). In addition, enhanced EV secretion may be mediated through the signaling of oncogenes such as mutant EGFR and RAS, the proto-oncogene SRC or loss of p53 function (82–85). Several ncRNAs have also been implicated in cancer EV secretion including lnc-HOTAIR which promotes transport of MVEs to the plasma membrane and exosome secretion by mediating Rab35 and SNAP23 localization (86). Lnc-HULC was reported to increase exosome secretion through a miR-372-3p/Rab11a axis (87). miR-200a was also found to target gelsolin and stabilize the polymerized actin networks to suppress MV secretion (88).

Vesicle trafficking is controlled by the Rab family of GTPases which cycle between an inactive GDP-bound and an active GTP bound state via GTPase-activating proteins and guanine nucleotide exchange factors, respectively. Each endosomal compartment is enriched in certain GTP-bound Rab proteins, which interact with specific effector proteins to regulate multiple steps of vesicle trafficking, including vesicle formation, cargo selection, movement, tethering/fusion with the plasma membrane and vesicle uncoating (89). The two Rab27 isoforms (RAB27A and RAB27B) have key functions that mediate the vesicle trafficking process (89). Although both RAB27 proteins can recruit the same effector proteins, RAB27A and RAB27B function in EV secretion appears to be non-redundant, regulating distinct steps (90). RAB27B mediates the transfer of MVBs from microtubules to the actin cortex and transport to the cell periphery whereas RAB27A is required for the tethering and docking of the MVBs to the cell membrane (90). Furthermore, knockdown of RAB27B results in perinuclear clustering of MVBs of decreased size, whereas knockdown of RAB27A results in a decrease in the number of MVBs formed (90). NSCLC CSCs have been reported to secrete elevated levels of EVs when compared to non-CSCs (52). This increase in EV secretion was found to be mediated by targeted upregulation of RAB27B in NSCLC CSCs. Furthermore, RAB27B dependent EV secretion was shown to be important for the maintenance of a stem-like phenotype in NSCLC cells, including spheroid growth, clonal expansion, transformed growth, invasion, tumor growth, and metastasis (52). Interestingly, compared to EVs derived from differentiated NSCLC cells, NSCLC CSC-derived EVs are preferentially internalized by differentiated NSCLC cells, and upon internalization, led to a RAB27B-dependent increase in stem marker expression, invasion, self-renewal, and transformed growth in these cells (52). These data suggest that RAB27B may be involved in regulating the cargo content of NSCLC CSC-derived EVs. Similarly, RAB27B is upregulated in CD34^+^ acute myeloid leukemia (AML) CSCs and to plays a role in the stemness of these cells (91). Mechanistically, RAB27B directly interacts with senescence-associated proteins in AML CSCs to promote their selective loading into secreted exosomes. In addition, AML CSC-derived exosomes enriched with senescent-associated proteins remodeled the CSC niche to induce senescence of mesenchymal stem cells (MSCs) in a RAB27B-dependent manner (91). These senescent MSCs in turn secreted exosomes enriched with stemness-promoting proteins to maintain stemness in AML cells. These data provide mechanistic insight into the role of RAB27B in regulating CSC-derived EV cargo and trafficking to drive cancer stemness.

### 3.3 CSC-derived EVs in NSCLC stemness, progression and metastasis

**Figure 2:**
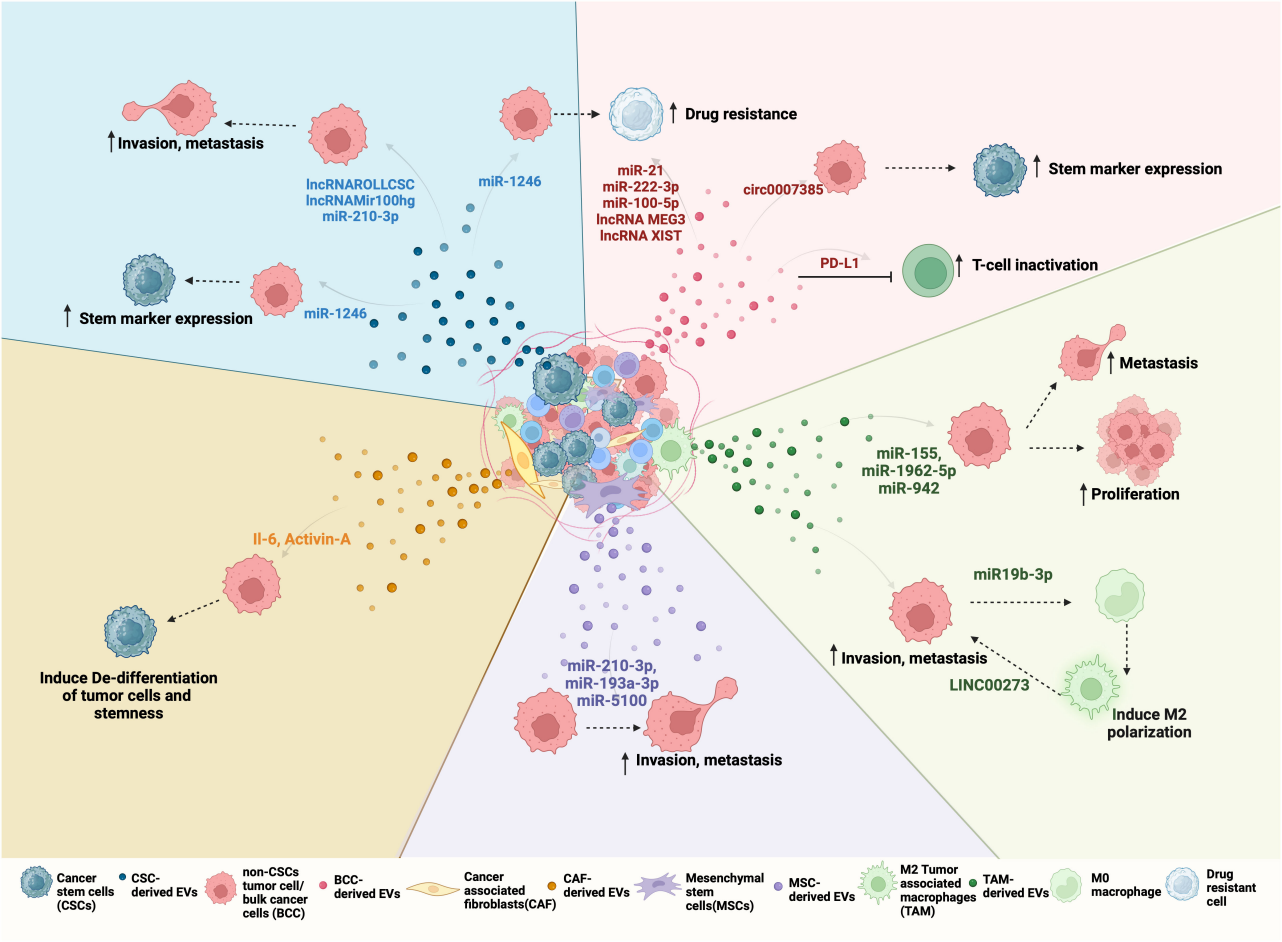
Bidirectional EV-mediated communication in NSCLC progression: NSCLC TME consists of different cell types such as differentiated cancer cells or bulk cancer cells (BCC), cancer stem cells (CSC), cancer-associated fibroblasts (CAFs), tumor-associated macrophages (TAMs), mesenchymal stem cells (MSCs). All of these cells can release EVs with unique cargo that is internalized by other cells of the tumor to regulate its behavior. For example, (clockwise right to left) Red EVs: Tumor-derived EVs or BCC-derived EV cargo, including miRNA, lncRNA, and circRNA can promote drug resistance and stemness. BCCs also secrete PD-L1-containing EVs that inactivate CD8+ T cells and promote drug resistance. Green EVs: TAMs, when polarized to M2, secrete EVs to promote proliferation and metastasis by transferring LINC000273 and reciprocally, BCC-derived EV cargo miR-19b-3p can induce M2 polarization. Purple EVs: MSCs-derived EV mediated transfer of miR-210-3p, miR-191-a and miR-5100 to BCC promote their invasion and metastasis. Orange EVs: CAFs can induce de-differentation and stemness by transferring IL-6 and Activin-A to BCCs. CAFs can also support CSC growth by secreting IGF11. Blue EVs: CSC-derived EVs carry ncRNA such as lnc-ROLLCSC, lnc-Mir100hg, miR-210-3p, and miR-21-5p which promote invasion, metastasis, and miR-1246 which promote stemness and drug resistance in BCCs.

CSC-derived EVs mediate many aspects of cancer progression including proliferation, angiogenesis, immune evasion, metastasis, and drug resistance. Since EVs can enter the circulation and potentially travel far from the site of their origin, they are widely investigated for their role in premetastatic niche formation and colonization of cancer cells at metastatic sites. Once the CSC-derived EVs enter the circulation, they can reach distant organs and release factors directly into the recipient cells, modifying their gene expression to mobilize cells that constitute the pre-metastatic niche (92). In addition to a role in establishing a premetastatic niche, CSC-derived EVs may contribute to NSCLC metastasis by inducing stemness in differentiated cancer cells or the acquisition of an EMT phenotype (93). Differentiated NSCLC cells internalize CSC-derived EVs more preferentially when compared to non-CSC-derived EVs (52). Furthermore, upon internalization, CSC-derived EVs induced stem marker expression and sphere-forming ability in differentiated cancer cells (52). The ability of NSCLC CSC-derived EVs to promote stem-like characteristics and EMT is mediated by a variety of biologically active EV cargo molecules, including non-coding RNAs (**Figure 2** *)*. For example, one study demonstrated that NSCLC derived-EVs transferred miR-210-3p to differentiated cancer cells which led to an increase in EMT markers such as N-cadherin, Vimentin, MMP-9, and MMP-1 and downregulated E-cadherin expression in these cells (94). Interestingly, EV delivered miR-210-3p targeted *FGFRL1* in the differentiated cancer cells, resulting in increased migration and invasion in these cells (94). Another study demonstrated the role of a novel lnc-ROLLCSC in CSC-derived EVs in modulating a stem phenotype in differentiated NSCLC cells (95). In this study, lnc-ROLLCSC was identified as the most significantly upregulated lncRNA in CSC-EVs in comparison to non-CSC-derived EVs. Furthermore, delivery of CSC-derived EV encapsulated lnc-ROLLCSC to differentiated cells could induce a more aggressive metastatic phenotype by targeting miR-5623-3p and miR-217-5p (95). In a similar study, the lnc-Mir100hg was shown to be upregulated in NSCLC CSCs (96). CSC-derived exosomes transferred Mir100hg to differentiated cancer cells which promoted an enhanced migratory and invasive phenotype through targeting of the tumor suppressor miRNAs, miR-15a-5p and miR-31-5p. EVs derived from brain metastasizing NSCLC cells carry oncogenic miR21 that facilitates EMT and metastasis in non-metastatic lung tumor cells (97). Additionally, non-metastatic cells treated with miR-21 containing EVs exhibited an increase in self-renewal, generated more tumor spheres, and were considered stem-like cells (97). miRNA-499a-5p is upregulated in both highly metastatic NSCLCs and their exosomes (98). Exosome delivered miRNA-499a-5p conferred proliferation, EMT, and migration properties to recipient NSCLC cancer cells through regulation of the mTOR pathway (98). Liu et al. isolated exosomes from NSCLC CSCs grown as spheres and upon treatment of parental A549 cells with these exosomes observed an increase in CSC markers (*OCT4*, *SOX2*, *NANOG*, *ALDH1*) as well as an increase in sphere number and size (38). Furthermore, they identified miR-1246 as significantly upregulated in CSC-derived exosomes, and miR-1246 targeted the *TRIMP17* transcripts in recipient differentiated NSCLC cells to promote their stemness (38). In addition to ncRNAs, specific EV protein cargo have been identified as mediators of a metastatic phenotype in NSCLC cells. CSC-derived EVs carry stemness marker proteins such as CD133, CD44, and NOTCH1 and elicit different responses in recipient cells when compared to non-CSC-derived EVs. For example, proteomic profiling of EVs isolated from highly metastatic and poorly metastatic lung cancer cells, identified HGF as specifically enriched in EVs of highly metastatic cancer cells (99). HGF secreted in metastatic cell EVs activated downstream c-Met signaling which induced proliferation, migration and invasion by promoting EMT signaling in recipient non-metastatic cells (99). Taken together, these studies demonstrate that NSCLC CSCs secrete EVs that carry cargo, which, when internalized by differentiated cancer cells, induce molecular changes to promote stemness, EMT and metastatic phenotypes in NSCLC.

Therapeutic resistance is a hallmark characteristic of CSCs. There is increasing evidence suggesting that EVs from drug-resistant cells can transfer specific cargo including drug resistance-related proteins, nucleic acids, and metabolites to neighboring drug sensitive cancer cells or directly package and sequester drugs out of donor cells, leading to the development of drug resistance. For example, A549 NSCLC parental cells treated with EVs secreted from A549 cis-platin resistant cells exhibited an increase in the activity of DNA damage response genes such as *BRCA1*, *ERCC1*, as well as miR-21 that mediated resistance to cis-platin (100). A similar study demonstrated that the EVs of cisplatin-resistant A549 NSCLC cells carry miR-100-5p that could confer cisplatin-resistance and reduced apoptosis in recipient A549 parental cells (101). The miR-222 family members have been implicated in EV-associated drug-resistance in lung cancer (102). Specifically, miR-222-3p was detected in EVs released from gemcitabine-resistant A549 which could induce gemcitabine-resistance in recipient sensitive A549 parental cells (102). Gefitinib-resistant EGFR T790M mutant PC9R cells were found to transfer prosurvival Akt/mTOR complexes in EVs that could stimulate proliferation, invasion and drug-resistance to gefitinib-induced apoptosis in parental PC9 cells (103). Kwok et al. reported that lnc-MEG3 and lnc-XIST are differentially expressed in EVs secreted by drug-resistant ALK-translocated NSCLC cells. These circulating EV-RNAs can induce drug resistance in other subpopulations in addition to maintaining intratumoral heterogeneity (104).

### 3.4 EV-mediated NSCLC CSC and TME bi-directional cross-talk

CSCs reside in highly specialized niches surrounded by differentiated cancer cells, and cells of the tumor microenvironment (TME) that include fibroblasts, endothelial, immune and mesenchymal stem cells (MSCs) (105). The CSC niche is designed to maintain and protect the CSCs and may also allow CSCs to stay dormant for long periods of time, before initiating local recurrent and/or distant metastatic tumors (106). Reciprocally, CSCs can modulate the TME to favor tumor progression and metastasis formation. Increasing evidence supports a role for EV-mediated mechanisms in the bidirectional interactions between NSCLC CSCs and cells of the TME (70, 71). EVs secreted by CSCs or stromal cells in the TME can transfer cargo molecules, including microRNA (miRNA), long noncoding RNA (lncRNA), and circular RNA (circ RNA) that modulate the niche and support CSC growth, stemness, invasion, metastasis, immune evasion, EMT and angiogenesis in NSCLC (**Figure 2**).

MSCs are pluripotent stromal cells that are chemotactically recruited from bone marrow (BM) to a tumor site by chemokines and cytokines originating from cancer cells (107). These BM-MSCs can evolve and differentiate based on the signals from cancer cells and TME to promote tumorigenesis. BM-MSCs can release various bioactive molecules that can influence stemness, drug resistance, or the maintenance of the CSC phenotype (108). For example, a study by Zhang et al. explored the role of exosomal miRNA secreted from BM-MSCs in promoting lung cancer metastasis (109). BM-MSCs grown under hypoxic conditions secrete exosomes containing miRNAs such as miR-193a-3p, miR-210-3p, and miR-5100 that when internalized by NSCLC cells activated the STAT3 pathway, leading to the upregulation of mesenchymal markers such as Vimentin, Twist, and SNAIL and promoted metastasis (109). In contrast to the tumor-promotive roles ascribed to MSCs, there are several studies demonstrating a tumor inhibitory role for MSC-derived EVs. For example, BM-MSC-derived exosomal miR-30b-5p inhibited tumor growth in mice by blocking a EZH2/PI3K/AKT signaling axis (110). A similar study reported that BM-MSC-derived exosomes can carry miR-144 which targets the expression of cyclin E1 (*CCNE1*) or cyclin E2 (*CCNE2*) and inhibit the NSCLC progression (111). BM-MSC derived exosomes are also involved in mediating chemosensitivity. Specifically, cis-platin resistant NSCLC cells showed decreased invasion, proliferation, colony formation, and increased apoptosis when treated with miR-193a containing exosomes derived from BM-MSCs (112, 113). How the balance between the tumor-supportive and suppressive roles of BM-MSCs is regulated is an interesting line of further investigation.

CSC-derived EVs can transform fibroblasts to cancer-associated fibroblasts (CAFs) that exhibit myofibroblastic differentiation and perform various functions that promote tumor progression and metastasis (114). CAFs in turn secrete EVs that can induce EMT or stemness in cancer cells. For example, Chen et al. reported the involvement of CAF paracrine signaling in the growth of CSCs (61). Specifically, they demonstrated that when CAFs from patient lung tumor tissue are co-cultured with CSCs, there is an increase in stem cell markers (*OCT3/4*, *NANOG*) and sphere forming ability of the CSC population. Interestingly, CSCs exhibit a decrease in anchorage-independent growth and *NANOG, OCT3/4* gene expression when grown without CAF feeder cells, suggesting the absence of CAFs led to differentiation of the CSCs (61). CAFs can induce de-differentiation of terminally differentiated lung carcinoma cells in co-cultures under stress conditions (20). These de-differentiated lung cancer cells exhibited stem-like characteristics such as increased sphere formation, upregulation of EMT marker (Vimentin), low doubling time, and resistance to methotrexate. The de-differentiation effects were traced to IL-6 carried in CAF derived-exosomes that activate JAK/STAT2 and downstream Notch/Wnt pathway signaling (20). These data suggest that fibroblasts, under tumor-promoting conditions, can secrete EVs that induce de-differentiation of tumor cells to gain cancer stem cell-like properties.

In the TME, both innate (tumor-associated macrophages (TAMs) and myeloid-derived suppressor cells (MDSCs)) and adaptive immune cells (Tregs) participate in regulating tumor activities (115). The communication between CSCs and these immune cells plays a significant role in the mechanisms that promote evasion of immune surveillance, a cancer characteristic that influences tumor progression. CSCs can produce factors secreted in EVs that drive TAM polarization and persistence of the TME in an immunosuppressive state. For example, EVs isolated from NSCLC CSCs have increased miR-21 levels that induced polarization of M2 TAMs which are associated with pro-tumor signals that increase cancer cell proliferation, invasion, and metastasis (116). Likewise, when NSCLC tumor cells and unpolarized macrophage cells are co-cultured, NSCLC cells induce polarization of macrophages by exosome secreted miR-19b-3p regulation of PTPRD/STAT3 signaling in the macrophage cells (117). Reciprocally, TAMs may promote EMT reprogramming and the growth of NSCLC CSCs. M2 TAMs, which are abundant in the metastatic tissues of NSCLC, secrete exosomes that promote tumor proliferation, migration, invasion, and EMT of NSCLC cells (118). Mechanistically, M2 TAM exosomes were found to be enriched in miR-155 and miR-1962-5p, which inhibits and negatively regulates a potential tumor suppressor gene Ras association domain family member 4 (*RASSF4*) (118). A similar study showed that miR-942 is transferred by M2 TAM-derived exosomes to the NSCLC cells and promotes NSCLC progression (119). M2-derived exosomes delivered miR-942 to NSCLC cells; miR-942 was then shown to bind a region of the FOXO1 3’-UTR that regulates β-catenin activation in NSCLC cells (119). Cancers cells may also acquire immunotolerance through inactivation of cytotoxic T cells (CD8+), a mechanism that can be mediated by EVs. NSCLC cells can secrete exosomes that contain PD-L1, which acts to promote NSCLC stemness and increased resistance to cisplatin (120). Interestingly, the co-culture of NSCLC cells with CD8^+^ T cells resulted in inactivation of CD8^+^ T cells in a secreted PD-L1-dependent manner (120).

In human NSCLC, neutrophils were shown to be the most abundant immune cell type in the TME (121). An analysis of infiltrating immune cells in lung adenocarcinomas revealed an association between tumor-associated neutrophil (TAN) signatures and poor disease outcomes, suggesting that neutrophils are an important component within the TME of lung tumors (122). Analogous to macrophages, TANs exhibit the ability to be polarized from an anti-tumorigenic (N1 phenotype) to pro-tumorigenic (N2 phenotype) subpopulations depending on the signals and interactions with other TME components (123). Consistent with these data, Eruslanov et al. reported that in early stages of lung cancer, TANs exhibit an N1 phenotype as measured by their immune-stimulating capabilities and ability to trigger an anti-tumor T-cell response (124). In contrast, N2 TANs have been reported to promote the tumorigenic and metastatic potential of lung cancer cells (125) as well as stimulate immunosuppression and angiogenesis (126). Mechanistically, TANs have been found to induce migration and invasion in lung cancer cells by inducing expression of *NOTCH3*, a marker of stemness (127). TANs have also been reported to secrete the cytokine IL-10 to facilitate the activation of c-Met/STAT3 signaling which promoted the distant metastasis of lung cancer cells (126). Interestingly, lung cancers in turn activated a STAT3/PD-L1 axis that facilitated the polarization of TANs towards the N2 phenotype (126) demonstrating crosstalk between the cancer cells and TANs. Furthermore, these data suggest a shift from N1 to N2 TANs as lung tumors progress. There is evidence to support that EVs may mediate the cross talk between cancer cells and TANs. For example, tumor cells were found to secrete EVs that carry snRNAs capable of activating Toll-like receptor-3 (TLR-3) in lung epithelial cells which induced chemokine secretion in the lung. This led to neutrophil recruitment to promote pre-metastatic niche formation for metastasis of tumor cells to the lung (128). Other studies have linked N2 neutrophils to the metastasis of lung cancer cells to the brain. Specifically, chronic nicotine exposure was shown to recruit N2 neutrophils to the brain pre-metastatic niche. These N2 neutrophils secreted EVs containing miR-4466 which promoted stemness and metastasis in lung tumor cells by activating a SKI/SOX2/CPT1A signaling axis (129). Interestingly, in colon cancer, stem-like cells secrete EVs containing RNA that induce interleukin-1B (*IL-1B*) expression and activate an NF-*κ*B signaling axis to increase neutrophil granulocyte lifespan and polarization of TANs to a pro-tumorigenic phenotype that promotes tumorigenesis of colon cancer (130). These data highlight the importance of EVs in CSC-neutrophil interaction during cancer progression. Whether this mechanism is also operative in NSCLC CSCs has not been investigated.

Endothelial cells, another vital component of the TME, play a significant role in promoting angiogenesis, which supplies nutrients required for tumor cell growth. Furthermore, formation of vasculature within the tumor facilitates cancer cell entry in the circulation, thus promoting metastasis. Studies on the role of EVs in angiogenesis have demonstrated that cancer cell-derived EVs contain interleukin-6 (IL-6) and vascular endothelial growth factor (VEGF), potent pro-angiogenic factors that enhance endothelial cell invasion and organization in tubule-like structures (131). Hypoxia and the HIF-1 signaling pathway have been implicated in maintaining the CSC phenotype (132). Under hypoxic conditions, NSCLC cells have been shown to release exosomes containing HIF-a and COX2, which promoted angiogenesis (133). NSCLC-derived EVs can also carry pro-angiogenic ncRNAs such as miR-23a, which stimulate angiogenesis and increase permeability in endothelial cells under hypoxic conditions (134). Endothelial cells can mutually support CSCs by for example, providing Notch ligands that promote Notch signaling to maintain a CSC phenotype (135) or induce the expansion of the CSC population by release of basic fibroblast growth factor (bFGF) (136). EVs released from human brain microvascular endothelial cells induced expression of S100A16 in lung cancer cells, which in turn promoted lung cancer cell survival and metastasis to the brain (137), further demonstrating the tumor-promoting effects of endothelial cell-derived EVs.

## 4 EVs as potential diagnostic and prognostic biomarkers for NSCLC

Biomarkers to allow early detection and monitoring of NSCLC progression, relapse, and therapeutic resistance are needed. NSCLC-derived EVs carry proteins, nucleic acids, lipids, and metabolites that have been well characterized for their roles in NSCLC progression, metastasis, and therapeutic resistance. Furthermore, cancer cell-derived EV cargo reflects the pathological state of cancer and exhibit distinct differences from the cargo of non-transformed cells. EVs are remarkably stable and can be detected in body fluids such as blood, plasma, serum, urine, sputum, and pleural effusions that are collected in the clinic. Thus, NSCLC secreted EVs represent a promising non-invasive tool that can be used in the development of diagnostic, predictive, and prognostic biomarkers for NSCLC.

### 4.1 Protein biomarkers

Proteins within EVs have emerged as potential NSCLC biomarkers (**Table 2**). For instance, EGFR localized on exosomal membranes can be used to differentiate NSCLC and chronic lung inflammation (138). This finding was corroborated by another study that identified EGFR along with its interacting proteins (GRB2, calmodulin, CD59, and RAB5), specifically in exosomes isolated from the pleural effusion of NSCLC patients. These studies emphasize the potential use of exosomal EGFR as a diagnostic biomarker for lung cancer (139). The analysis of serum exosomes from lung cancer patients identified CD91 as a NSCLC diagnostic marker with higher sensitivity for detecting stage-I and II LUAD patients than the classical clinical biomarker carcinoembryonic antigen (140). Leucine-rich a2-glycoprotein (LRG1), found at elevated levels in urine and lung tissue-derived exosomes of NSCLC patients, emerged as another candidate diagnostic marker for NSCLC (141). Another study conducted by Jeong et al. reported that exosomal GCC2 levels associated with NSCLC pathological stage, and demostrated that exosomal GCC2 may be used as an early-stage marker (142).

**Table 2:** Summary of potential diagnostic, predictive and prognostic biomarkers in NSCLC derived EVs.

Classification	Biomarker	Source	Key findings	Reference
Protein	EGFR	Serum	Differentiate NSCLC and chronic lung inflammation	(138, 139)
	CD91	Serum	LUAD specific antigen on exosomes	(140)
	LRG1	Urine	Potential urinary biomarker	(141)
	GCC2	Serum	Associated with progression of NSCLC pathological stage	(142)
circRNA	circSATB2	Serum	Associated with lung cancer metastasis	(143)
	circUSP	Serum	May predict response to anti-PD1 therapy	(144)
lncRNA	UFC1	Serum	UFC1 levels associated with NSCLC invasiveness	(145)
	DLX6-AS1	Serum	Associated with advanced disease stage, metastasis, and poor differentiation	(146)
	RP5-977B1	Serum	Elevated in early-stage NSCLC cases, and associates with poor prognosis	(147)
	MLETA1	Serum	Exosomal lnc-MLETA1 levels correlate with metastasis in lung cancer patients	(148)
microRNA	let-7f	Serum	Associated with NSCLC patient survival, distinguish between early and late-stage patients	(149)
	miR-30e-3p	Serum	Associated with NSCLC patient survival distinguish between early and late-stage patients	(149)
	miR-1290	Serum	Serum exosomal levels demonstrated superior diagnostic efficiency over clinical markers, CEA, CYFRA21–1, and NSE in discriminating between NSCLC patients and healthy controls.	(150)
	miR-378	Serum	Levels decreased after radiation treatment; marker for radiotherapy response	(151)
	miR-146a-5p	Serum	Role in cisplatin resistance. Serum exosomal miR-146a-5p levels associated with NSCLC recurrence	(152)
	miR-200 family	Pleural effusion	Identified patients with NSCLC from individuals with benign lung disease	(153)
	miR-21	Pleural lavage	Associated with NSCLC invasion in the pleural cavity pre-metastatic niches formation	(154)
Other	LCN2 (mRNA)	Pleural effusion	Identified patients with NSCLC from individuals with benign lung disease	(153)
	cfDNA	Plasma	EV-DNA present in advanced stages of cancer	(155)
	Lipid	Plasma	Distinguished between early- and late-stage NSCLC patients from normal subjects	(156)

### 4.2 RNA biomarkers

EV cargo contain different species of ncRNA, such as circRNA, lncRNA, and miRNA, all of which have been reported to be potential diagnostic or prognostic biomarker candidates for NSCLC (**Table 2**). circSATB2 promotes NSCLC progression and is upregulated in serum exosomes collected from lung cancer patients. Serum exosome circSATB2 levels were highly selective in lung cancer patients with metastasis, suggesting its potential use as a biomarker for NSCLC progression (143). Exosomal circUSP secreted by NSCLCs inhibits CD8^+^ T cell function and contributes to anti-PD1 immunotherapy resistance (144). Thus, exosomal circUSP could be a useful marker to predict response to anti-PD1 therapy. Similar roles have been demonstrated for EV lncRNAs as potential biomarkers for NSCLC progression. Elevated levels of the lnc-UFC1 were detected in serum exosomes of NSCLC patients, and increased UFC1 levels were associated with NSCLC invasion (145). Distal-less homeobox 6 antisense RNA 1 (DLX6-AS1) is upregulated in NSCLC tumor tissues and cell lines and associates with advanced disease stage, metastasis, and poor differentiation (146). DLX6-AS1 was found in serum exosomes of NSCLC patients and showed a higher sensitivity for diagnosing NSCLC than CYFRA21-1, a known serum diagnostic marker of NSCLC (146). Another novel lncRNA, RP5-977B1 has elevated levels in patient serum exosomes, even in early-stage cases, and associates with poor prognosis suggesting lnc-RP5-977B1 is a promising diagnostic and prognostic marker for NSCLC (147). Additionally, the exosomal lnc-MLETA1 has been implicated in promoting migration and invasion of lung cancer cells. Furthermore, exosomal lnc-MLETA1 levels correlate with metastasis in lung cancer patients making it a potential prognostic marker correlated with patient metastasis (148).

Several studies have provided valuable insights into the potential use of plasma EV-derived miRNAs as biomarkers for NSCLC (**Table 2**). For example, the analysis of EVs from the plasma of NSCLC patients and controls identified a panel of 10 miRNAs (has-let-7, has-mir-223, has-mir-383, has-mir-345, has-mir-192, has-mir-301, has-mir-20b, has-mir-572, has-mir-30e-3p, and has-let-7f) that were differently packaged (149). Validation of these miRNAs on an expanded cohort of NSCLC patients and controls revealed that expression levels of let-7f, miR-30e-3p were significantly decreased in the plasma EVs of NSCLC patients were associated with a higher rate NSCLC patient survival (149). Furthermore, the levels of let-7f and miR-30e-3p were able to distinguish patients who underwent surgical resection (stage I,II, and IIIA) and those who did not (stages IIIB and IV), suggesting the potential role of these EV secreted miRNAs in informing NSCLC treatment decisions based on disease stage (149, 157). A similar study demonstrated differences in the serum exosome levels of miR-1290 of NSCLC and healthy patients (150). In a ROC analysis involving 90 patients, exosomal miR-1290 demonstrated superior diagnostic efficiency compared to CEA, CYFRA21–1, and NSE in discriminating between NSCLC patients and healthy controls (150). miR-378, identified as another potential biomarker linked to poor survival in NSCLC was reported to be decreased after radiation treatment, suggesting its potential use as a marker for radiotherapy response (151). Likewise, exosomal miRNA miR-146a-5p levels were found to decrease in the process of cisplatin induced resistance in NSCLC cells, and serum exosomal miR-146a-5p levels associate with NSCLC recurrence, indicating serum exosomal miR-146a-5p may be a potential tool to monitoring cisplatin resistance in NSCLC patients (152). In addition to serum, several studies have examined EVs isolated from pleural effusion and observed differences in EV miRNA and mRNA content of patients with NSCLC and benign inflammatory diseases. Notably, the miR-200 family and Lipocalin-2 (LCN2) mRNA in exosomes could stratify patients with lung adenocarcinoma from individuals with benign lung disease, suggesting that these two RNAs may serve as potential diagnostic markers for the NSCLC (153). The presence of tumor cells in pleural lavage is associated with tumor recurrence and poor outcomes in NSCLC (154). High EV‐miR‐21 expression level in pleural lavage fluid was associated with NSCLC invasion in the pleural cavity, suggesting a potential use for miR-21 as a diagnostic marker with implication for pre-metastatic niches formation (154). Taken together, these findings underscore the value of EVs in serum and pleural effusion for liquid biopsy and provide insights into NSCLC diagnosis and prognosis. These findings also highlight the promise of analyzing specific ncRNAs from NSCLC patient plasma EVs for non-invasive diagnosis, stratification based on disease stage, and prediction of long-term outcomes in NSCLC.

### 4.3 Other markers

The processes underlying the active release of DNA by tumor cells remain elusive, particularly regarding how circulating cell-free DNA (cfDNA) are selectively sorted into EVs. An interesting study conducted by Abe et al. analyzed cfDNA in the plasma of lung cancer patients, revealing the simultaneous presence of longer fragments of DNA with EVs in advanced stages of cancer, while shorter fragments were not found within the same EVs (155). This suggests that EVs might shield the longer DNA fragments from degradation and that these fragments could potentially serve as biomarkers. Despite these promising findings, there is still a need for further exploration to comprehensively understand the complex interactions between DNA and EVs, as well as the clinical implications of different EV DNA cargo in NSCLC. EVs are enclosed in lipids, and these various lipids which include sphingolipids, phosphatidylserine, and cholesterol can reflect EV biogenesis and may provide insights into the activation of various stress, metabolic, inflammation, or pathologic programs and responses. Fan et al. investigated lipid profiles in plasma exosomes for early detection of NSCLC (156). They identified 16 lipids with promising performance in distinguishing between early- and late-stage NSCLC patients from normal subjects, highlighting the potential of EV lipid analysis for detecting NSCLC at different stages (156).

## 5 EV-based therapeutic strategies for targeting NSCLC

Compelling evidence indicates that EV-mediated communication between NSCLC CSCs, differentiated cancer cells, and the TME mediate the mechanisms that promote NSCLC tumor initiation, progression, metastasis, immune evasion, therapeutic resistance, and tumor relapse. Therefore, EV-mediated signaling represents an important target for developing therapies for NSCLC. Here, we have highlighted three potential therapeutic strategies: 1) inhibition of EV biogenesis and secretion, 2) targeting of EV molecules and their cargo, and 3) engineering of EVs with specific cargo to target pathways important for the stemness of NSCLC (**Table 3**).

**Table 3:** Summary of EV-based therapeutic strategies targeting NSCLC.

Strategy	Inhibitor	Target	Therapeutic Effect	Reference
Inhibition of EV biogenesis and secretion	PPIs such as Omeprazole	proton pumps	EV retention; decreased tumor cell proliferation	(158)
	GW4869	Neutral Sphingomyelinase	Decreased EV secretion and EMT marker expression. Combined treatment with EGFR-TKI reduced tumor burden	(159)
	Ketoconazole	Rab27A	Disrupted EV trafficking and blocks cancer cell growth	(160)
Targeting of EV molecules and their cargo	Antibodies	CD9, CD63 EV surface proteins	Decreased breast cancer cell metastasis to the lungs	(161)
	NMT1 inhibitors	protein palmitoylation	Inhibited the loading of Src into EVs	(162)
EVs engineering with specific cargo	PSiNPs-encapsulated doxorubicin	CD44+ CSC population	Reduction of CSC population in subcutaneous, and metastatic tumor	(163, 164)
	EV-encapsulated lnc-LOC85009	ATG5-induced autophagy	Increased NSCLC cells sensitivity to docetaxel.	(165)
	EVs-encapsulated miR-7-5p	MAPK/eIF4E signaling	Suppressed NSCLC growth by increasing sensitivity to an mTOR inhibitor	(166)

EV-mediated communication may be inhibited at the level of biogenesis and secretion. For example, exosomal trafficking is regulated by microenvironmental pH and proton pump inhibitors, which can induce acidification of the tumor cell cytosol can cause EV retention (158). GW4869, a non-competitive inhibitor of neutral sphingomyelinase (nSMase2) has been well characterized for its function as an inhibitor of exosome secretion (158). Combined treatment of GW4869 and gefitinib (EGFR-TKI) in mice significantly reduced the growth of tumors (159). In addition to inhibiting EV secretion, GW4869 was shown to inhibit the expression of EMT markers in NSCLC cells (159), suggesting that GW4869 may target EV directed signals in stemness phenotypes such as EMT. Another strategy to inhibit EV biogenesis is to target factors that regulate EV trafficking within cells such as the use of ketoconazole to target RAB27A, which is required for MVB formation, resulting in block of tumor cell growth (160). Since EVs are secreted by almost all cells of the body, more studies will be needed to assess the long-term effects and toxicity of targeting EV biogenesis or EV secretion. The tumor cells might rely on multiple vesicle types such as MVs or ectosomes to mediate cell-cell communication. Thus, targeting a specific class of EVs based on the biogenesis, might have a short-term effect and give rise to potential resistance to inhibitors of EV secretion.

A second approach is to target EVs or their cargo that play important roles in mediating intercellular communication. For example, monoclonal antibodies against EV markers CD9 and CD63 targeted circulating EVs of breast cancer cells in a mouse xenograft model (161) leading to a significant decrease in metastasis to the lungs (161). In addition, EV cargo that are important for mediating signaling between CSCs and the TME maybe targeted directly through genetic or pharmacological inhibitors. For example, myristoylation and palmitoylation of the SRC family kinases including SRC and FYN facilitates their packaging into EVs (162). Interestingly, genetic loss or pharmacological inhibition of these fatty acylation modifications inhibited the loading of SRC and FYN into EVs (162).

EVs are actively being investigated for their application as drug delivery systems to inhibit tumor growth. EVs are naturally generated by cells, stable in body fluids, biocompatible, less immunogenic/toxic and, importantly, they can cross the blood-brain barrier (167). Thus, there are many benefits of using EVs as a therapeutic tool. There is a growing interest in engineering EVs to enhance their targeting and cargo delivery capabilities to inhibit CSCs and NSCLC tumor growth and progression (167). These engineered EVs can be loaded with miRNA/siRNAs or pharmacologic agents that will target molecules involved in stemness to inhibit tumor growth. Yong et al. have developed biocompatible tumor-cell derived exosome-biomimetic porous silicon nanoparticles referred to as PSiNPs to carry and deliver chemotherapy drugs such as doxorubicin (DOX) (163). Interestingly, exosome-encapsulated DOX accumulate in the side population (CSCs) of tumors *in vivo*, which resulted in the reduction of CSC population in subcutaneous, orthotopic, and metastatic tumor models (163). To specifically target NSCLC CSCs, EVs can be artificially engineered to target surface markers such as CD133, CD44 and CD166. For example, anti-CD44 antibody coated EVs packaged with doxorubicin were used to target the CD44^+^ CSC population in colon carcinoma (164). Targeting of CSC surface markers may be challenging since these markers are also expressed by normal stem cells and not all CSCs express the same surface markers. Therefore, targeting more than one surface marker may improve the efficacy and specificity of this targeting strategy. EVs are also being explored for the delivery of cargo that reduces cancer cell drug resistance. For example, exosomal delivery of lnc-LOC85009 to docetaxel-resistant NSCLC cells increased their sensitivity to docetaxel-mediated apoptosis (165). Another study demonstrated that tumor suppressor miR-7-5p regulates MAPK/eIF4E signaling, which is involved in regulating sensitivity to the mTOR inhibitor everolimus (166). Exosome delivery of miR-7-5p was shown to synergize with everolimus in blocking the growth of NSCLC cells (166). The use of EVs as a vehicle to deliver chemotherapy agents or tumor-suppressive molecules to target tumor cells holds great promise. However, more studies that focus on the engineering the EVs to encapsulate molecules of different sizes or developing drugs to be used as cargo to inhibit important CSC processes are needed.

## 6 Concluding remarks

CSCs play critical roles in the mechanisms that drive NSCLC lethality and treatment failures, suggesting that NSCLC CSCs must be effectively targeted in order to elicit effective and lasting therapeutic benefits in NSCLC patients. Bidirectional, EV-mediated signaling between CSCs and the various other cell populations within NSCLC tumors, has gained interest due to the roles these signals play in NSCLC invasion, metastasis, cell proliferation, stemness, and therapeutic resistance. Proteins, RNAs, and lipids within EVs offer valuable insights into the complex mechanisms underlying NSCLC progression (**Table 2**). The cargo of EVs reflects the characteristics of the donor cell and pathological states such as NSCLC. Furthermore, EVs can be non-invasively isolated from bodily fluids. Thus, EVs have a strong potential to be developed as therapeutic targets and diagnostic, predictive and prognostic biomarkers. However, the development of EVs and their cargo as biomarkers may be hampered by a variety of technical challenges. For example, there needs to be more standardization of EV isolation, storage, and downstream analyses of patient sample-derived EVs for diagnosis in clinical settings. Since EV cargo reflects the molecular characteristics of the parental cell, the source of EVs (serum, plasma, and urine) might contribute to the heterogeneity of the EVs in the samples. Thus, for developing an EV-based biomarkers, it is important to consider the source and isolation method. Various studies have identified specific EV biomarkers such as EGFR, circSATB2, and miRNAs, and demonstrated their therapeutic implications and potential for early detection, and prognosis of NSCLC (138, 139, 143, 150–154). However, relying on individual biomarkers may not achieve optimal diagnostic performance while a combination of biomarkers may demonstrate enhanced efficacy. While challenges and complexities persist, the evolving landscape of EV research in NSCLC provides a foundation for future investigations and the development of innovative diagnostic and therapeutic strategies.

## Author contributions

PP: Conceptualization, Software, Visualization, Writing – original draft, Writing – review & editing. DA: Conceptualization, Visualization, Writing – original draft, Writing – review & editing. SK: Software, Visualization, Writing – review & editing. VJ: Conceptualization, Funding acquisition, Investigation, Project administration, Resources, Supervision, Writing – review & editing.
